# Evaluation of Emerging Predictors for Contrast-Induced Nephropathy in High-Risk Patients Undergoing Percutaneous Coronary Intervention

**DOI:** 10.7759/cureus.64363

**Published:** 2024-07-11

**Authors:** Himanshu Gupta, Mohit Mohan Singh, Krishna Kumar Sahani, Ayushi Gupta, Ganesh Seth

**Affiliations:** 1 Cardiology, Medanta Heart Institute, Medanta Hospital, Lucknow, IND; 2 Cardiology, Apollomedics Super Speciality Hospital, Lucknow, IND; 3 Anesthesia, Hind Institute of Medical Sciences, Safedabad, IND

**Keywords:** risk factors, prevention, nephropathy, contrast media, angiography

## Abstract

Objective: The objective of this study is to investigate the occurrence and factors that influence the development of contrast-induced nephropathy (CIN) in high-risk patients undergoing angioplasty and to evaluate the effectiveness of the Mehran risk score in predicting CIN among this patient population.

Materials and method: This prospective, observational study enrolled patients undergoing elective coronary angiography or a percutaneous coronary intervention (PCI) procedure. The patients were stratified into four risk groups based on the Mehran risk score, a validated tool for predicting the risk of CIN. Univariate and multivariate analyses were conducted to evaluate the risk factors associated with the development of CIN. A p-value of <0.05 was considered to indicate statistical significance.

Results: During the study period, a total of 55 high-risk patients underwent PCI. The incidence of CIN was 25.5% (n=14). Univariate and multivariate analyses revealed that age >75 years and estimated glomerular filtration rate (eGFR) <60 (p<0.05) were independently associated with a significantly increased risk of developing CIN. A considerable proportion of patients (23; 41.8%) in the study were categorized as having an intermediate risk for CIN based on the Mehran risk score.

Conclusion: This study observed a high incidence of CIN and encourages the use of predictive tools like the Mehran risk score to assess the risk of CIN occurrence, with age over 70 years and eGFR less than 60 emerging as significant.

## Introduction

Contrast-induced nephropathy (CIN) is defined as an acute impairment of kidney function that occurs after exposure to contrast agents used in radiological procedures. Various studies have reported the incidence of CIN to range between 5% and 25% [1,2]. CIN is currently the third leading cause of acute kidney injury (AKI) acquired during hospital stays, accounting for 10% of all cases of hospital-acquired acute renal failure [3]. It is associated with substantial illness and increased risk of death. Despite advancements in cardiac catheterization techniques and equipment improvements, researchers have faced challenges in mitigating this significant complication. Although progress has been made in interventional cardiology procedures and device technology, effectively preventing and managing contrast-induced kidney injury remains an ongoing struggle within the scientific community [4]. The risk of developing CIN is influenced by several factors, such as advanced age, exposure to large volumes of contrast material, pre-existing kidney dysfunction, diabetes mellitus, and hypertension. These patient characteristics and clinical factors have been associated with an increased likelihood of experiencing CIN [5]. The incidence of CIN tends to be higher following primary percutaneous coronary intervention (PCI) procedures [6]. Even with the implementation of preventive measures, approximately 20-30% of patients who have pre-existing risk factors still develop CIN [7-9]. Promptly and accurately identifying CIN is critical for preventing further deterioration and improving patient outcomes [6]. However, the utilization of risk scoring systems can provide valuable assistance in evaluating the likelihood of developing CIN within a given patient population. While several risk scoring models have been formulated based on the primary risk factors associated with contrast-induced nephropathy, the literature lacks comprehensive validation of any single risk score as an adequate predictive tool for this condition [10]. Sgura et al. have mentioned that the Mehran CIN risk score can be used to predict CIN development and aids in discriminating between low-, medium- and high- and very high-risk patient subgroups for consequent poor clinical outcomes during both short- and long-term follow-up [11]. No large studies to date looked at the risk predictors of CIN.

The study aimed to investigate the frequency and factors influencing CIN occurrence among high-risk patients undergoing coronary angioplasty. Additionally, we seek to assess the effectiveness of the Mehran risk score in predicting CIN within our specific patient demographic.

## Materials and methods

Study design

This was a prospective, observational study conducted at the Department of Cardiology for six months in India.

Inclusion criteria

Patients undergoing elective CAG/CAG+PCI procedures have been selected based on the presence of at least two risk factors including age (>65 years), diabetes, anemia (<10gm), recent ACS, LV dysfunction (EF<50%) or hypotension (SBP<90), congestive heart failure, and history of AKI/chronic kidney disease (CKD).

Exclusion criteria

Patients undergoing routine hemodialysis or peritoneal dialysis and patients whose eGFR was <15 ml/min were excluded from the study. The eGFR was calculated using the Cockroft-Gault formula.

A total of 55 patients were enrolled in the study based on the predetermined inclusion criteria. All participants received standard prophylactic measures to prevent CIN. These measures included continuous intravenous saline infusion starting 12 hours before and continuing for 24 hours after the PCI procedure, as well as the discontinuation of nephrotoxic medications. Iodinated contrast agents, specifically Omnipaque and Visipaque, were utilized during the procedures. For patients with heart failure, the rate of saline infusion was reduced to 0.5 ml per kilogram of body weight per hour to avoid over-hydration. Serum creatinine levels and eGFR were assessed before and after the procedure, as well as prior to hospital discharge. Various outcomes, including the incidence of CIN, delays in hospitalization, and the need for nephrology consultation, were evaluated.

Definitions

CIN

CIN was defined as an increase in serum creatinine concentration of 0.5 mg/dl (44mol/L) or 25% above baseline within 48 hours after contrast administration [4].

Maximum permissible contrast volume

Cigarroa et al. have established a validated upper limit for contrast usage in preventing CIN during PCI, expressed as follows: Divide five times the body weight in kilograms by the serum creatinine level in mg/dl [12].

In this study, the use of contrast was very limited ranging from 50ml to 100ml due to high risk of developing CIN among the patients. CIN is directly proportional to the amount of contrast used but we didn't find such association due to restricted contrast used and limited sample size.

Statistical analysis

The statistical analysis was performed with R software version 4.3.3 (the R foundation, Vienna, Austria). Continuous variables were summarized using mean ± standard deviation, while categorical data were presented as frequencies and percentages. A univariate analysis was conducted to identify potential risk factors associated with the development of CIN. Variables with a p-value less than 0.2 in the univariate analysis were then included in a multivariate analysis model. The results of the multivariate analysis were reported as odds ratios (ORs) with corresponding 95% confidence intervals (CI). Throughout the analysis, a two-sided p-value of 0.05 was considered statistically significant.

## Results

During the study period of six months, 55 high-risk patients who underwent CAG and CAG+PCI were prospectively evaluated for the development of CIN. The baseline characteristics of the population are demonstrated in Table 1.

**Table 1:** Baseline demographics of the patients ACS: Acute coronary syndrome; AKI: acute kidney injury; CKD: chronic kidney disease; LVEF: left ventricular ejection fraction; MI: myocardial infarction

Patient’s characteristics	No. of patients (n=55)
Age (years, mean ± SD)	62.18 ± 9.76
Male, n (%)	46 (83.6)
Weight (kg, mean ± SD)	69.71 ± 11.73
Height (cm, mean ± SD)	162.16 ± 8.11
Diabetes mellitus, n (%)	41 (74.5)
Hemoglobin (g/L, mean ± SD)	12.26 ± 1.99
Acute MI / ACS, n (%)	38 (69.1)
LVEF (%, mean ± SD)	46.43 ± 10.13
Systolic blood pressure (mm Hg, mean ± SD)	127.96 ± 19.34
Diastolic blood pressure (mm Hg, mean ± SD)	74.22 ± 8.37
Congestive heart failure, n (%)	13 (24.5)
History of AKI/CKD, n (%)	11 (20)

**Figure 1:**
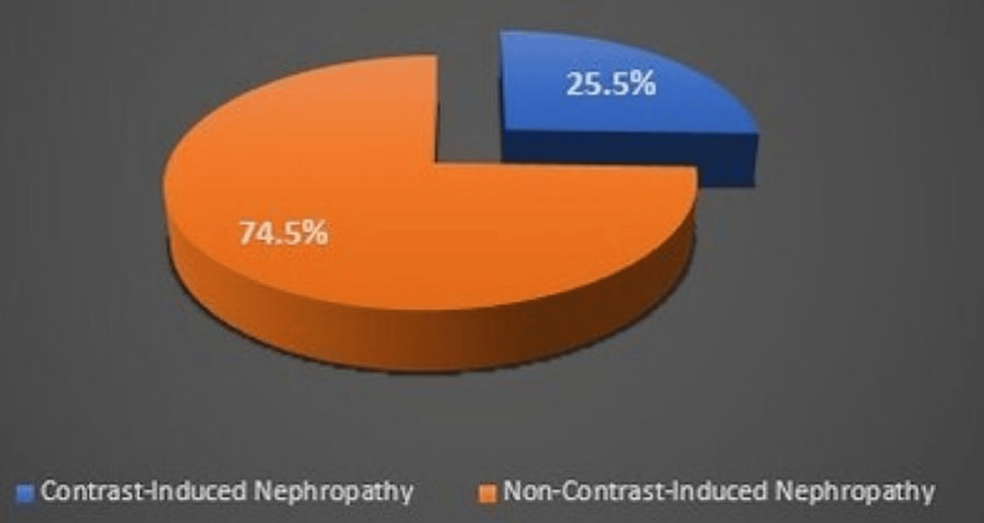
The pie diagram showing the incidence of contrast-induced nephropathy in patients after percutaneous coronary intervention

The study population had a mean age of 62.18 ± 9.76 years, with a majority being male patients. Of the patients studied, 41 (74.5%) had diabetes mellitus, while 13 (24.5%) were diagnosed with congestive heart failure. Out of the 55 high-risk patients who underwent assessment for the development of CIN, 14 patients (25.5%) experienced CIN during the study period. This is illustrated in Figure 1. Table 2 outlines the procedural specifications.

**Table 2:** Procedural details of the patients eGFR: Estimated glomerular filtration rate; NAHCO_3_: sodium bicarbonate; NAC: n-acetyl cysteine; NS: normal saline; RFT: renal function test

Variables	No. of patients (n=55)
Pre-procedure RFT	
Blood urea (mg/dl, mean ± SD)	39.45 ± 24.41
Serum creatinine (µmol/L, mean ± SD)	1.18 ± 0.57
eGFR (ml/min, mean ±SD)	75.14 ± 38.27
Pre-procedure assessment	
Fasting (hour, mean ± SD)	5.45 ± 2.28
Hydration (NS/NAHCO3), n (%)	31 (56.4)
NAC, n (%)	12 (21.8)
Contrast type and volume	
Omnipaque, n (%)	36 (65.5)
Visipaque, n (%)	19 (34.5)
Contrast volume (ml, mean ± SD)	95.20 ± 14.548
Post-procedure	
Hydration, ml/hr for 12-24 hours, n (%)	37 (67.3)
Hydration, ml/hr for 12-24 hours, (mean ± SD)	1465.45 ± 622.17
Serum creatinine	
After 24 hours (mean ± SD)	1.03 ± 0.45
After 48 hours (mean ± SD)	1.14 ± 0.38
eGFR on discharge (ml/min, mean ± SD	81.25 ± 40.58
Contrast Induced Nephropathy, n (%)	14 (25.5)

Among the patient’s cohort, 21 (38.2%) underwent multi-vessel PCI, while only 4 (7.3%) received single-vessel PCI. The average serum creatinine levels were 1.03±0.45 µmol/L after 24 h post-procedure and 1.14±0.38 µmol/L after 48 h. Additionally, the estimated eGFR at discharge was calculated to be 81.25±40.58. The Mehran risk score among patients is shown in Table 3.

**Table 3:** Patients distribution based on the Mehran risk score MRS: Mehran risk score

MRS Score	Risk Category	Patients, n (%)	Predicted risk of CIN from Mehran
≤5	Low	21 (38.2%)	7.5%
6-10	Intermediate	23 (41.8%)	14.0%
11-15	High	9 (16.4%)	26.0%
≥16	Very High	2 (3.6%)	57.3%

This study evaluates the accuracy of the Mehran risk score in predicting the occurrence of CIN among the patients included in our cohort. The majority of patients were classified as intermediate risk, based on their Mehran risk score. Specifically, 23 (41.8%) of the study population had scores between 6 and 10, placing them in this risk category. Within this group, there was 14% risk of developing CIN. On the other hand, 21 (38.2%) were classified as low-risk. These individuals had a Mehran risk score of 5 or lower, corresponding to a predicted risk of CIN of 7.5%. Notably, only a small proportion of patients i.e. 2 (3.6%) were classified as very high risk, with scores exceeding or equal to 16, yet this group demonstrated a significantly elevated risk of CIN, at 57.3%. In the distribution of predicted patient numerous across Mehran subcategories based on scores, half of the population in the very high-risk category developed CIN, while 6 (28.6%) of those in the low-risk category experienced the condition.

The traditional risk factors for CIN including anemia, age over 75 years, left ventricular ejection fraction, eGFR, diabetes mellitus, low blood pressure, and high blood pressure were analyzed among the population. A univariate analysis was conducted for these variables. Those variables with a p-value less than 0.2 in the univariate analysis were then included in a multivariate analysis model. From the multivariate analysis, advanced age and reduced eGFR emerged as statistically significant predictors for the development of CIN among the patients. Tables 4, 5 characterized the univariate and multivariate analysis for the dependent variable CIN with predictors.

**Table 4:** Univariate analysis for the dependent variable CIN with predictors ACS: Acute coronary syndrome; eGFR: estimated glomerular filtration rate; LVEF: left ventricular ejection fraction; MI: myocardial infarction; SBP: systolic blood pressure; CIN: contrast-induced nephropathy

Risk factors for the development of CIN	No. of patient n=55	p-value	OR (95% CI)
Age > 75, n (%)	6 (10.9)	0.028	7.8 (1.246-48.822)
Male, n (%)	46 (83.6)	0.555	1.591 (0.34-7.44)
Female, n (%)	9 (16.4)		
Weight (kg, mean ± SD)	69.71 ± 11.73	0.127	1.041 (0.989-1.096)
Height (cm, mean ± SD)	162.16 ± 8.11	0.914	1.004 (0.931-1.083)
Diabetes mellitus, n (%)	41 (74.5)	0.312	0.506 (0.135-1.895)
Haemoglobin (g/L, mean ± SD)	12.26 ± 1.99	0.171	1.262 (0.904-1.761)
Acute MI / ACS, n (%)	38 (69.1)	0.827	1.161 (0.306-4.402)
LVEF (%, mean ± SD)	46.43 ± 10.13	0.323	1.035 (0.967-1.107)
Systolic blood pressure (mm Hg, mean ± SD)	127.96 ± 19.34	0.666	0.989 (0.942-1.039)
Diastolic blood pressure (mm Hg, mean ± SD)	74.22 ± 8.37	0.64	0.973 (0.867-1.092)
Congestive Heart Failure, n (%)	13 (24.5)	0.385	0.479 (0.091-2.518)
Hypotension (SBP <80 for ≥1 hour requiring inotrope or balloon pump within 24 hours of Cath), n (%)	5 (9.1)	0.77	0.712 (0.073-6.961)
Anaemia (Baseline haematocrit value <39% for men and <36% for women), n (%)	9 (16.4)	0.301	0.317 (0.036-2.795)
eGFR < 60, n (%)	21 (38.2)	0.046	0.193 (0.038-0.973)

**Table 5:** Multivariate analysis for the dependent variable CIN with predictors eGFR: estimated glomerular filtration rate; CIN: contrast-induced nephropathy

Risk factors for the development of CIN	No. of patient n=55	p-value	OR (95% CI)
Age > 75, n (%)	6 (10.9)	0.030	9.728 (1.252-75.594)
Weight (kg, mean ± SD)	69.71 ±11.73	0.986	1.001 (0.940-1.065)
Haemoglobin (g/L, mean ± SD)	12.26 ± 1.99	0.783	1.054 (0.725-1.532)
eGFR < 60, n (%)	21 (38.2)	0.045	0.163 (0.028-0.957)

## Discussion

CIN poses a significant risk for high-risk patients undergoing PCI, a fact often overlooked. Our study brought attention to this danger, revealing an incidence of 14 (25.5%) among our study population, underscoring the high-risk profile of these patients.

Risk factors for CIN

In our investigation, 6 (10.9%) of the patient cohort exhibited an age exceeding 75 years old. Our analysis, both univariate and multivariate, identified age as a significant risk factor for CIN. This finding is consistent with the research by Chong et al. They similarly highlighted that individuals with advanced age, specifically surpassing 70 years, as a predictive factor for CIN [13]. One of the factors contributing to the risk of CIN development is reduced EF. In this study, we observed a mean LVEF of 46.43%, which was not statistically significant as a predictor of CIN (p=0.323). However, Ari et al. noted that patients with an ejection fraction below 40% faced an elevated risk of CIN [14]. The presence of low EF in patients is associated with diminished renal perfusion and the administration of numerous pharmacological agents, which may explain the heightened susceptibility to CIN. Out of the patients enrolled, 9 (16.4%) were diagnosed with anemia. Anemia emerged as a crucial risk factor significantly associated with CIN development. The presence of ionic contrast agents increases the binding affinity of hemoglobin to oxygen molecules, thereby impeding the delivery of oxygen to the metabolically active renal medullary region. The presence of anemia exacerbates hypoxic renal injury compounding its severity. Furthermore, for patients with CKD, every 3% decrease in hematocrit substantially elevates the odds of experiencing [15,16]. Valappil et al. [4] observed that 52% of the patients in their study had anemia, with a corresponding incidence of CIN of 44% within this subgroup. In our population, history taking revealed that 11 (20%) of individuals had a previous occurrence of AKI and CKD. Notably, the presence of CKD before PCI emerged as a significant risk factor for CIN incidence with deteriorating kidney function correlating with an escalated risk [17]. Additionally, independent predictors of CIN include diabetes and heart failure, conditions which are often comorbid with CKD [18,19]. Within our study cohort, 13 patients presented with congestive heart failure while 41 had diabetes. A total of 41 patients (74.5%) had diabetes and 9 (22%) among the diabetics had CIN. According to the literature, individuals with diabetes and elevated baseline creatinine levels are identified as having the highest risk of developing CIN [20,21]. In a study, it was discovered that individuals with insulin-dependent diabetes experienced a significantly higher incidence of CIN in comparison to diabetics managing their condition with oral hypoglycemic agents or dietary control [13].

Risk prediction scores for CIN

The Mehran risk score, proposed by Mehran et al. has been well-analyzed in various patients for predicting the risk of CIN in patients undergoing procedures involving radiocontrast media [2]. The Mehran risk score provides an evaluation of the specific risk for requiring hemodialysis treatment, stratified by each defined risk category. In our study, we observed a strong association between an increased Mehran risk score and the development of CIN. Patients belonging to the very high-risk group according to the Mehran risk score had a 24 times greater risk of developing CIN compared to those in intermediate and low-risk groups. Notably, 50% of the patients predicted to be at very high risk by the Mehran risk score were subsequently diagnosed with CIN. Consistent with the findings of Mehran et al., a significant proportion (41.8%) of the patients in our study fell into the intermediate-risk category. These results further validate the utility of the Mehran risk score in stratifying the risk of CIN and the subsequent need for hemodialysis in patients undergoing radiocontrast procedures [2,22].

Prophylactic measures

The first and most important step in renal protection is risk stratification and determination of the necessity of contrast administration. Despite extensive research on various treatments for kidney protection, only intravenous hydration with normal saline or sodium bicarbonate has proven effective in reducing CIN in at-risk individuals [23]. National and international guidelines, along with the expert consensus, recommend hydration strategies for both prevention and treatment of CIN [24]. A meta-analysis of seven clinical trials involving 2,851 patients confirmed that prophylactic hydration lowers the risk of CIN [25]. In our study, we implemented prophylactic measures, which may explain why only 14 out of all patients were diagnosed with CIN. In a retrospective study, research found that hydrating patients after a procedure was just as effective as hydrating them before it. They also suggested that postoperative hydration might expand volume effectively, reducing the chances of developing CIN [26]. Literature also suggests that injecting contrast into an artery tends to lead to a higher risk of CIN compared to injecting it intravenously. This could be because the concentration of contrast in the artery is higher after injection. Additionally, arterial procedures might cause further kidney damage, such as from atheroemboli [23]. All patients included in the study received intravenous hydration both before and after their procedure, taking into consideration the risk associated with intra-arterial administration.

The study was limited to a single center and focused on assessing the prevalence and factors of CIN specifically in high-risk patients undergoing PCI. Notably, parameters such as Cystatin C and kidney injury molecule-1, which are additional indicators for post-PCI renal dysfunction, were not incorporated in this investigation. Consequently, further trials involving larger cohorts are warranted to validate and extend the findings of this study.

## Conclusions

In conclusion, our study observed a high incidence of CIN and encouraged the use of predictive tools such as the Mehran risk score to assess the risk of CIN occurrence. Age over 70 years and eGFR less than 60 emerged as significant factors contributing to this risk. Unlike other studies, our findings did not associate anemia or LVEF as predictive risk factors. These results emphasize the significance of targeted monitoring and interventions for individuals at risk.
